# The impact of artificial intelligence in medicine on the future role of the physician

**DOI:** 10.7717/peerj.7702

**Published:** 2019-10-04

**Authors:** Abhimanyu S. Ahuja

**Affiliations:** Charles E. Schmidt College of Medicine, Florida Atlantic University, Boca Raton, FL, United States of America

**Keywords:** Artificial intelligence, Machine learning, Impact, Medicine, Physician, Deep learning, Radiology, Pathology, Opthalmology, Oncology, Cardiology

## Abstract

The practice of medicine is changing with the development of new Artificial Intelligence (AI) methods of machine learning. Coupled with rapid improvements in computer processing, these AI-based systems are already improving the accuracy and efficiency of diagnosis and treatment across various specializations. The increasing focus of AI in radiology has led to some experts suggesting that someday AI may even replace radiologists. These suggestions raise the question of whether AI-based systems will eventually replace physicians in some specializations or will augment the role of physicians without actually replacing them. To assess the impact on physicians this research seeks to better understand this technology and how it is transforming medicine. To that end this paper researches the role of AI-based systems in performing medical work in specializations including radiology, pathology, ophthalmology, and cardiology. It concludes that AI-based systems will augment physicians and are unlikely to replace the traditional physician–patient relationship.

## Introduction

The term “Artificial Intelligence” (AI) was first coined by John McCarthy for a conference on the subject held at Dartmouth in 1956 as “the science and engineering of making intelligent machines” (Society for the Study of Artificial Intelligence and Simulation of Behavior, 2018). After a period of reduced funding and interest in AI research, also referred to as the AI winter (Crevier, 1993), optimism in AI has generally increased since the low point in the early 1990s. Artificial intelligence (AI) is an important field of computer science that seeks to create complex machines with characteristics of human intelligence. We can think of this concept as “General AI,” which has machines that can think and reason and even see and hear like humans (Copeland, 2016). This concept which can be seen in movies like Star Wars (think C-3PO, a droid programmed for etiquette and protocol) is not something we can achieve at this time. However, what is achievable at this time falls under the concept of “Narrow AI” where technologies exist to perform specific tasks as well as, or better than, humans can (Copeland, 2016). Examples of such narrow AI include speech recognition, facial recognition, etc. These technologies exhibit certain facets of human intelligence. Such intelligence is derived from AI techniques known as machine learning and deep learning which have improved performance in areas such as image classification, text analysis, speech and facial recognition with a range of promising applications such as autonomous vehicles, natural language processing, and in medicine.

AI is poised to play an increasingly prominent role in medicine and healthcare because of advances in computing power, learning algorithms, and the availability of large datasets (big data) sourced from medical records and wearable health monitors. The health care market for AI is increasing at a rate of 40% and is expected to reach $6.6 billion by 2021 (Frost & Sullivan, 2016). Computing power is increasing rapidly due, in part, to the wide availability of Graphics Processor Units that make parallel processing even faster and the availability of seemingly infinite compute resources on demand in the cloud. Big data is also well supported by practically endless storage in the cloud. Learning algorithms are becoming more precise and accurate as they interact with training data, allowing newer insights into diagnostics, treatment options, and patient outcomes (Bresnick, 2018b). The flood of health care data is helping push the development of new AI applications that promise to improve the efficiency and effectiveness of patient care. Healthcare related big data is available from sources such as Electronic Medical Records (EMR) and wearable health trackers, which can be analyzed in new ways. The rise of AI in the era of big data can assist physicians in improving the quality of patient care and provide radiologists with tools for improving the accuracy and efficiency of diagnosis and treatment. AI is well-suited to handle repetitive work processes, managing large amounts of data, and can provide another layer of decision support to mitigate errors. The research firm Frost & Sullivan estimates that AI has the potential to improve patient outcomes by 30% to 40% while reducing treatment costs by up to 50% (Hsieh, 2017a).

Experts predict AI to have a significant impact in diverse areas of health care such as chronic disease management and clinical decision making (Bresnick, 2016). While still in the early stages of adoption, AI algorithms are showing promise in specializations such radiology, pathology, ophthalmology, and cardiology (Hsieh, 2017a). This progress raises a thought-provoking question. Will AI at some point displace certain physicians such as radiologists or will it help make them more effective or will it be a bit of both? This research looks at the potential uses of AI in medicine and considers the possibility of AI replacing certain physicians or at least supplementing the role of physicians.

The rest of this paper is organized as follows. A survey of the literature is provided in ‘Literature Survey’. ‘Artificial Intelligence vs. Machine Learning vs. Deep Learning’ provides a discussion on AI, Machine Leaning, and Deep Learning and how they relate to each other. ‘Promise of AI in Modern Medicine’ discusses the promise of AI in medicine across various specialties such as radiology, pathology, cardiology, and ophthalmology. ‘Assessing the Impact of AI on Physicians’ assesses the impact of AI on physicians. Conclusions are provided in ‘Conclusions’.

### Survey methodology

Scholarly articles that were reviewed in this paper were searched in journal databases such as PubMed and subject-specific professional websites including Google Scholar. The search terms that were used when searching for articles included artificial intelligence, medicine, machine learning, deep learning, radiology, pathology, cardiology, oncology, and ophthalmology. Inclusion criteria for selected articles required that articles be directly related to the topic on artificial intelligence and medicine. Both qualitative and quantitative articles were reviewed. Qualitative articles provide insights into the problem by helping understand reasons, opinions, and motivations. Quantitative articles on the other hand use measurable data to formulate facts and discover patterns in research.

## Literature Survey

Patel et al. (2009) present the findings of a panel discussion held at an AI in Medicine (AIM) conference in 2007 in the Netherlands in a position paper titled, “The coming of age of artificial intelligence in medicine”. The authors reflect on the maturity of AI research and its impact on the field of medicine and attempt to characterize the influence of AI in medicine thus far. The authors opine that one indication of the success of AI in medicine is that AIM methods are becoming more integrated within applications and are not explicitly visible as such. The question being asked has moved from “does the system work?” to “does the system also help?” and this, according to the authors, indicates that AIM-based solutions are being implemented in clinical practice (Patel et al., 2009). According to the authors, machine learning research is becoming more important for the future because medicine is now a data-rich quantitative field due to the increasing prevalence of Electronic Medical Records (EMR) and such research is important to make sense of this data and to create discovery-support systems that support the work of a human physician (Patel et al., 2009).

King Jr (2018) discusses the impact of AI on the field of radiology. Radiology has witnessed tremendous strides with the advent of ultrasound, Computed Tomography (CT), Magnetic Resonance Imaging (MRI), and Positron Emission Tomography (PET) scanning technologies. The author believes that the next breakthrough will come not from some new scanner technology but as a courtesy of AI utilizing the imaging data that is already available from imaging technologies such as ultrasound, CT, MRI, and PET (King Jr, 2018). According to the author, AI in radiology is likely to appear in stages with the initial stage already making an appearance. In this first stage AI systems perform automatic segmentation of various structures within CT and MR images (King Jr, 2018). This will help isolate and identify pathologic lesions for analysis and will yield significant savings in time for radiologists (King Jr, 2018).

Recht & Bryan (2017) discuss the impact on radiologists as machine learning/artificial intelligence is more integrated into clinical practice. According to the authors, AI will become a routine part of radiologists’ lives and make their work more efficient and accurate (Recht & Bryan, 2017). Per the authors, it is a possibility within the next 10 years that medical images will first be pre-analyzed by an AI tool before being reviewed by a radiologist. The tool would perform routine reading tasks such as quantification, segmentation and pure pattern recognition (Recht & Bryan, 2017a).

Kruskal et al. (2017) provide a summary of the deliberations at the 38th Radiology Intersociety Conference held in 2016. The participants discussed applications of machine learning to image analysis which they consider to be potentially disruptive. Though the initial reaction of radiologists to the automated interpretation of images may not be welcoming, the radiologists overall had a sense of optimism about the future of radiology with the machine learning based technologies augmenting the quality of care (Kruskal et al., 2017). Radiologists, it was felt, should remain active in the development of these new technologies by engaging with industry to ensure the ethical usages and clinical relevance of developing technologies (Kruskal et al., 2017).

Greenspan, Van Ginneken & Summers (2016) focus on technology based on deep learning as it applies to medical imaging. According to the authors deep learning has emerged as the leading machine-learning tool in general imaging. Convolutional neural networks (CNNs) have been shown to be very effective in the field of computer vision (Greenspan, Van Ginneken & Summers, 2016). Deep CNNs automatically learn mid-level and high-level abstractions obtained images and recent results show that CNNs are extremely effective in object recognition and localization in natural images (Greenspan, Van Ginneken & Summers, 2016).

Quantitative analysis of brain MRI depends on accurate segmentation of structures of interest. Deep learning algorithms can now be trained with millions of images, in part due to the rapid increase in Graphical Processing Unit processing power (Akkus et al., 2017). The authors in Akkus et al. (2017) summarize that while deep learning techniques in quantitative brain MRI has made big strides, “it is still challenging to have a generic method that can deal with all variations in brain MR images from different institutions and MRI scanners”.

In the article, “Your Future Doctor May Not be Human,” Norman (2018) postulates that the role of AI in healthcare is not necessarily about pitting human minds against machine. Rather, “AI is in the exam room to expand, sharpen, and at times ease the mind of the physician so that doctors are able to do the same for their patients” (Norman, 2018). The role of AI in medicine is expanding from diagnostic algorithms to surgical robots. What is unknown currently is the value of AI in medicine and the author cites several examples where AI is already just as capable, if not more than capable, as doctors in diagnosing patients (Norman, 2018). Norman states that AI is perhaps most valuable in medicine when making sense of huge amounts of data that would quickly overwhelm humans. The author concludes that the role of AI in medicine isn’t about replacing physicians; rather it is about optimizing and improving what they already do (Norman, 2018).

Bertalan Meskó, also known as The Medical Futurist, has called AI the “The Stethoscope of the 21st Century” (Meskó, 2017). According to the author, just as it took time for the medical community to accept the stethoscope, so it will also take the medical community time to accept AI as a full-fledged tool for healthcare (Meskó, 2017). Even so, Meskó predicts that AI will take its rightful place in healthcare with cognitive computers helping physicians in clinical decision making. In order to make this a reality, Meskó recommends that physicians should learn how AI works in healthcare, companies such as IBM should increase awareness in the general public about the advantages and risks of using AI in medicine, and healthcare institutions should measure the effectiveness of the AI-based system (Meskó, 2017).

Zaidi (2018) highlights application areas of AI in healthcare that look to be very promising. One of these areas is cognitive assisted robotics which is considered minimally invasive whereby large incisions are replaced with a series of quarter-inch incisions and utilize miniaturized surgical instruments. Furthermore, surgeons integrate the data from patient pre-op medical records with real-time operating metrics to improve surgical outcomes (Zaidi, 2018). Case in point is the da Vinci Surgical System which is a surgical system with robotic limbs which provides a high-definition, magnified, 3-D view of the surgical site (Zaidi, 2018). It facilitates complex surgery using a minimally invasive approach and is controlled by a surgeon from a console. On the administrative side of healthcare, Gohil discusses AI applications that automate non-patient care activities such as writing chart notes, prescribing medications, ordering tests. Such AI-based systems that depend on machine learning help healthcare providers cut documentation time and improve reporting quality (Zaidi, 2018).

Sennaar (2018) discusses the emerging role of AI in the hospital setting. One of the factors leading to burnout among physicians is managing the growing amounts of clerical and clinical data and AI applications can help in this regard. One of the risks identified by the author has to do with data quality. Poor data quality or “bad data” may contribute to inaccurate clinical data potentially putting patients at risk (Sennaar, 2018). According to the author, healthcare systems already possessing an effective data collection strategy may be better suited to implement AI-based applications for data management. The author also touches upon factors which may cause resistance in the adaptation of AI-based applications. This includes the limited amount of scientific literature demonstrating the clinical value of AI applications (Sennaar, 2018).

Houssami et al. (2017) discuss the promise and current reality of AI in breast cancer (BC) detection. While mammography screening for BC can confer a mortality benefit through early BC detection, it can also miss a cancer that is present, or can result in false-positives. To try and improve results mammography screenings have mostly focused on additional imaging in the form of double instead of single-reading and more frequent screens (Houssami et al., 2017). Such additional imaging increases resource expenditures. According to the authors AI can assist mammography screenings become more efficient by advanced learning using large complex datasets and by performing tasks such as image interpretation. The authors conclude that while AI is very promising for BC screenings, to translate this promise into reality requires very large data-sets of imaging examinations that are associated with clinical factors to train AI models (Houssami et al., 2017).

Another article on BC detection by Trister, Buist & Lee (2017) points out that digital mammography is an imperfect tool with a sensitivity of 84% for detecting breast cancers. The other 16% are not detected due to a combination of factors. This has a lot to do with the human limitation of what radiologists are visually able to identify on mammographic images (Trister, Buist & Lee, 2017). False positives on the other hand lead to significant anxiety, biopsies, and unwarranted surgical removal of tissue. The authors report that critics of screening have suggested that a significant percentage of screen-detected breast cancers constitute overdiagnosis or cases that would not have become clinically significant during women’s lifetimes (Trister, Buist & Lee, 2017). Clearly this is an important issue to women’s health and is an area where AI can help. The authors report that AI algorithms in digital mammography have converted single whole digital images of the breast into automatically extracted quantitative, pixel-level variables which are unrecognizable to the human eye (Trister, Buist & Lee, 2017). Computers can cluster these millions of pixel-level variables to identify new imaging features associated with BC, which can be associated with outcome data for training (Trister, Buist & Lee, 2017). The ultimate promise is that AI can combine these pixel-level variables and associations with patient clinical data, including any known patient risk factors, to develop predictive algorithms that may someday provide equal or better accuracy than human screening mammography (Trister, Buist & Lee, 2017).

A Letter to the Editor article in the European Journal of Internal Medicine touches upon the impact of AI on physicians (Krittanawong, 2018). The author writes that processing healthcare related big data offers the promise of unlocking new insights and accelerating breakthroughs in medicine which in turn can help improve clinical practice. It is very time consuming at present for physicians to analyze big data and requires sophisticated analytic tools. AI can assist physicians in analyzing such big data and help to improve the quality of patient care (Krittanawong, 2018). Physicians employ personal medical histories, physical exams, individual biomarkers, and scores such as CURB-65 (severity score that estimates mortality of community-acquired pneumonia) to diagnose patients (Krittanawong, 2018). AI on the other hand can assist physicians by diagnosing diseases using “complex algorithms, hundreds of biomarkers, imaging results from millions of patients, aggregated published clinical research, and thousands of physician’s notes from EMRs” (Krittanawong, 2018).

Liew (2018) discusses the need for the safe implementation of AI systems in radiology, where radiologists are mandatory as component human authorities. This is referred to as ‘radiologist-in-the-loop’ (Liew, 2018). In this scheme of things radiologists would be needed to lead multidisciplinary meetings, make judgement calls, and verify reports. According to the author, machine learning in the form of image processing, computer vision and natural language processing are the key AI technologies which would augment radiology in the future (Liew, 2018). The expectation here is that this would lead to a lowering of the overall cost of imaging to the patient by increasing the productivity of radiologists by automating time-consuming and low cognitive value tasks.

Roach (2017) looks at some of the applications of AI in Ophthalmology. The most promising application is in the detection of retinal eye diseases. The author reports that researchers from the Google Brain project had reported in 2016 that the deep learning AI system had taught itself to accurately detect diabetic retinopathy (DR) and diabetic macular edema in fundus photographs (Roach, 2017). AI is also being applied to other retinal diseases such as age-related macular edema (AMD). According to the article, AI-based systems are likely to be used by ophthalmologists as just another tool in their tool set to diagnose eye diseases (Roach, 2017) and to improve the sensitivity to detect patients at risk and the specificity to correctly identify those without disease.

## Artificial Intelligence vs. Machine Learning vs. Deep Learning

AI is a subfield of Computer Science that deals with the design and development of intelligent machines (Society for the Study of Artificial Intelligence and Simulation of Behavior, 2018). It deals with the use of computers to mimic the cognitive functions of humans and carry out tasks based on algorithms in an intelligent manner such as learning from experience, adjusting to new inputs, and performing human-like tasks (Venkatesan, 2018). AI has been used in medicine before. Expert systems, an earlier form of AI, tried to encode the decisions of physicians into a set of rules that computers could execute (Schmidt-Erfurth et al., 2018). It was, however, not possible to encode a set of rules for all clinical situations given the complexity of medicine and variations in diseases. For these reasons expert systems were replaced in the 1990s by Machine Learning (ML) where the “rules would be learned by algorithms directly from a set of examples instead of being encoded by hand” (Schmidt-Erfurth et al., 2018).

ML is a subset of AI and emphasizes the learning aspect of intelligence. It has to do with developing computer programs that can learn and improve from experience without being explicitly programmed. This stands in contrast to traditional computer programs which require specific instructions that detail every step the program must take. It would not be incorrect to say that today when we refer to AI we have almost exclusively ML and its derivatives in mind. ML research continues to reference the brain as an inspirational source and attempts to “imitate the neural structure of the nervous system by creating artificial neural networks (ANNs) which are networks of units called artificial neurons organized into layers” (Pearson, 2017). The system learns to detect patterns from data provided to the system in a training session. The ML approach requires that a set of features be directly measured from the data (e.g., size of breast lesions in CT scans).

A deep neural network (DNN) is an ANN consisting of more layers (usually more than five) that allows for improved predictions from data. A significant advantage of DNNs is that their performance continuously improves as the size of the training dataset increases (Schmidt-Erfurth et al., 2018). This started a new subfield of ML called deep learning which employs algorithms such as DNN and convolutional neural networks (CNNs). The idea is “that a neural network, instead of just acting as a classifier as in the case of classic ML, can also function as the feature extractor as well” (Ahuja & Halperin, 2019). This allows for end-to-end training because a DNN learns to recognize an output category from the input signal directly (Schmidt-Erfurth et al., 2018). CNNs are mostly applied to image segmentation and classification. Graphical Processing Units enable significant acceleration in the case of CNNs (about 40 times) compared to CPU processing alone (Schmidt-Erfurth et al., 2018).

## Promise of AI in Modern Medicine

As pointed out earlier, several factors have come together recently to support the quickening pace of AI developments in medicine (Pratt, 2018). These include the amount of healthcare data collected in recent years, the high-level computing power at low cost now available to process large datasets, the increasing prevalence of EMRs, and overall advances in computing technologies, which have all fueled AI’s advancements in medicine (Pratt, 2018). While AI in medicine is still in its early stages, it is well positioned to make positive impacts in clinical medicine. As an example, AI could collect and analyze patient data gathered from multiple sources such as fitness trackers and at-home monitors and enable physicians to monitor patients’ health in ways that time and resources without AI would not permit (Pratt, 2018). Some of the specializations in medicine where AI is having a positive impact include radiology, pathology, ophthalmology, and cardiology. This section discusses the impact and potential of AI in these specializations.

### Diagnostic radiology

AI’s, and specifically ML’s, potential to analyze large datasets and extract meaningful insights is proving helpful in both radiology and pathology. Images from MRI machines, CT scanners, and X-rays can contain large amounts of complex data that can be difficult and time consuming for human providers to evaluate (Kent, 2018). AI/DL technology and its implementation into day-to-day clinical imaging is poised to transform the practice of radiology (Liew, 2018). AI can provide clinical decision support to radiologists and improve the delivery of care to patients. With regard to image processing, DL algorithms can help select and extract features from medical images as well as help create new features. With respect to image interpretation, DL algorithms can help identify and classify disease patterns from images and help the radiologist suggest suitable care pathways for a patient in consultation with other physicians involved in the care of the patient.

A research study by Lakhani & Sundaram (2017) used DL algorithms in the diagnoses of tuberculosis (TB) in chest X-ray images and achieved high levels of accuracy. The researchers obtained 1,007 X-rays of patients with and without active TB. The datasets were split into training (68.0 percent), validation (17.1 percent), and test (14.9 percent). The cases were used to train two different deep convolutional neural network (DCNN) models—AlexNet and GoogLeNet—which learned from TB-positive and TB-negative X-rays (Lakhani & Sundaram, 2017). The models’ accuracy was tested on 150 cases that were excluded from the training and validation datasets. The best performing AI model, a combination of the AlexNet and GoogLeNet, achieved a net accuracy of 96 percent which is comparable with human radiologists. It is likely that this can be improved with additional training cases and more advanced deep learning models (Lakhani & Sundaram, 2017).

Kooi et al. (2017) compared a traditional state-of-the art in mammography Computer Aided Detection (CAD) system which relied on a manually designed feature set with a DL Convolutional Neural Network (CNN) algorithm to detect breast cancer lesions. Both systems were trained on a large dataset of approximately 45,000 images and results showed that the CNN outperformed the traditional CAD system (Kooi et al., 2017). According to the authors, the system based on DL was shown to perform at the level of a radiologist.

With respect to radiology, AI is poised to automate image segmentation, lesion detection, measurement, labelling and comparison with historical images (Liew, 2018). The one thing worth pointing out is that DL, and indeed any ML, algorithms are only as good as the training sets used to train the system. The quantity and quality of the training set are very important in the development of state-of-the art DL systems in radiology (Lugo-Fagundo et al., 2018). In the case of CT scans, this requires the careful annotation of hundreds or even thousands of images. The creation of well-annotated training sets for this study is extremely labor intensive (Lugo-Fagundo et al., 2018). The authors in (Lugo-Fagundo et al., 2018) suggest that institutions develop standard processes and best practice guidelines for training datasets and share these datasets. Such collaborations would help increase the number of normal and abnormal datasets available for training DL systems.

### Pathology

The field of pathology depends on the trained eye of the pathologist to render a diagnosis of a biospecimen (Dyche, 2018). Given the many different types and subtypes of a disease and the avalanche of new data in the form of different biomarkers and genomics data, this is becoming an increasingly difficult task for the pathologist. In addition, fewer of the nation’s senior medical students choose to pursue a career in pathology (Dyche, 2018). In this scenario DL based approaches have a significant role to play. For example, researchers at Google trained a DL based CNN to assist with the detection of metastatic breast cancer in lymph node tissue on specimen images with an accuracy comparable to that achieved by human pathologists (Hsieh, 2017). It is clearly a challenging task to look for very small deposits of cancer on a specimen slide and a human pathologist can get fatigued, but an AI-based system suffers from no such problem and can scan any number of specimen slides with no loss of accuracy due to fatigue. Couple this with the expected amount of big data in the form of human genomic data, human pathologists will find it nearly impossible for pathologists to stay current with the emergence of new these biomarkers without the help of ML (Dyche, 2018).

On a different note, ML has been shown to accurately predict how long a kidney will function adequately in patients with chronic kidney damage. Specifically, a research team at Boston University used renal biopsies to train DL based CNN to predict kidney function. The researchers found that CNN algorithms “were more precise and accurate than traditional pathologist-estimated scoring systems when calculating kidney decline” (Bresnick, 2018a). Clearly, AI-based systems have the potential to augment clinical decision making for nephrologists.

### Ophthalmology

Many Ophthalmology practices are already using ML and DL to revolutionize vision care. The immediate impact has been observed in the field of retinal diseases. An AI-based device has already been FDA approved to detect diabetic retinopathy (FDA News Release, 2018). Schlegl et al. (2018) developed a deep learning-based system to “automatically detect and quantify intraretinal cystoid fluid (IRC) and subretinal fluid (SRF). This system accurately characterized the pattern of intraretinal fluid in patients with wet AMD or retinal vein occlusion (RVO) and distinguished between intraretinal cysts and subretinal fluid” (Schlegl et al., 2018). The authors conclude that deep learning in retinal image analysis provides an accurate means “for the differential detection of retinal fluid types across the most prevalent exudative macular diseases and OCT devices” (Schlegl et al., 2018).

AI systems are also being used beyond retinal diseases. “AI systems are being developed and evaluated to diagnose and grade cataract in pediatric patients based on an analysis of slit-lamp images (Liu et al., 2017), diagnose glaucoma based on measurement of the retinal nerve fiber layer (RNFL) thickness and visual field (VF) (Kim, Cho & Oh, 2017), and diagnose keratoconus based on Scheimpflug tonometry” (Ruiz et al., 2017). It is becoming more important that an ophthalmologist learn about the use of AI.

### Cardiology

A research study in the UK showed that ML can improve cardiovascular risk prediction by correlating complex interactions between risk factors (Weng et al., 2017). The researchers provided data on 295,000 patients to four ML algorithms (random forest, logistic regression, gradient boosting machines, neural networks) for training purposes for correlating medical history with heart attack rates. Next, the algorithms were made to predict which of additional 82,000 patients would have heart attacks based on their records (Hsieh, 2017; Weng et al., 2017). The best performing ML algorithm, neural networks, accurately predicted 7.6% more events than the American College of Cardiology/American Heart Association (ACC/AHA) method with 1.6% fewer false alarms (Hsieh, 2017a). For the given test sample size of approximately 83,000 records, this correlates to 355 more patients whose lives could have been saved (Hsieh, 2017a). The authors concluded that “Machine-learning significantly improves accuracy of cardiovascular risk prediction, increasing the number of patients identified who could benefit from preventive treatment, while avoiding unnecessary treatment of others” (Weng et al., 2017).

Another study by Dawes et al. (2017) developed a ML algorithm that enabled them to predict outcome in patients with pulmonary hypertension with high accuracy. Medical data from 250 patients were used for the study. According to the authors the ML algorithm “improved survival prediction independent of conventional risk factors in patients with newly diagnosed pulmonary hypertension” (Dawes et al., 2017).

### Looking to the future

While predicting the future of AI in medicine is no easy task it can certainly be said that AI has a role to play in medicine as a partner. This assessment is based on certain characteristics of AI-based systems. Systems such as IBM’s Watson can search through millions of pages of data, read countless medical articles, and far exceed the capacity of any human physician in its breadth and scale of knowledge (Sappin, 2018). An overworked physician may forget that a certain patient is vulnerable to a certain drug’s side-effect, but an AI-based system will not. AI can also assist in surgery in combination with augmented reality programs.

We know that one particular strength of AI-based systems is their ability to gather and analyze mountains of data and draw conclusions from its analysis. This can help in predicting disease, which is a challenging task for human physicians in the best of cases. AI can assist the physician in assessing cancer risk or assessing the risk factors of heart attacks as compared to strokes (Sappin, 2018). In the foreseeable future physicians could then help their patients preempt these conditions with a long-term plan of patient care (Sappin, 2018). All these changes are poised to improve the practice of medicine in the future. This will stem from the practice of medicine becoming more accurate, more comprehensive, and possibly less expensive over time by preempting diseases, and reducing harmful side-effects, and unnecessary tests based on patient profile. This is clearly good news for all concerned, patients, their families, and health care providers.

### Transforming medical care

In combination with a human physician AI methods and systems can advance the delivery of care in a way that outperforms what either can do alone. Consider the case of precision medicine which seeks to tailor medical treatment to the individual characteristics of a patient. This is likely to transform the delivery of medical care by enabling physicians to identify ideal medication dosages, determine which genetic mutations drive certain cancers, and sequence our microbiome. Central to making precision medicine possible is AI and ML which can make sense of massive amounts of clinical, genomic, and imaging data, thus helping to improve physician efficiency, increase diagnostic accuracy, and personalize treatment.

AI-based systems are poised to increase diagnostic efficiency in other areas of medicine as well. According to Pearl (2018), such AI-based systems will have a positive impact on various diagnostic fields including “radiology (CT, MRI and mammography interpretation), pathology (microscopic and cytological diagnoses), dermatology (lesion evaluation for potential melanoma), and ophthalmology (retinal vessel examination to predict the risk for diabetic retinopathy and cardiovascular disease).” According to the American Cancer Society about half of the women getting annual mammograms over a 10-year period will have a false-positive finding (American Cancer Society, 2017). False-positive mammograms can cause anxiety and often lead to unnecessary tests such as MRIs, ultrasounds, and even invasive biopsies to be sure cancer isn’t there, which cost time and money, and physical discomfort (Griffiths, 2016).

Another area where AI-based systems are expected to have an impact is in robotic surgery. Robotic surgery can assist surgeons in treating patients with precision, decreased blood loss, and less pain (Kakar, 2017). It reduces the possibility of tissue trauma because the robotic system lowers grasping forces due to tactile feedback and provides the surgeon with opportunity for remote surgery (Kakar, 2017; Wottawa et al., 2016). Robots enabled with AI can help reduce surgeon variations that could affect patient recovery.

Wearable healthcare devices (collectively referred to as Healthcare Internet of Things) from fitness trackers to portable blood pressure and insulin monitors will enable remotely managed healthcare by directly feeding data not only into individual treatment plans and EMRs, but also into larger AI-assisted analytics systems (Singh, 2018). This will allow for at-home management and monitoring of acute conditions, allowing physicians to make better treatment decisions (Singh, 2018).

### Case study: assisting oncologists in India using IBM Watson

IBM’s AI-based Watson for Oncology, a cognitive computing platform trained by Memorial Sloan-Kettering Cancer Center that analyzes data to identify evidence-based treatment options, has been adopted by Manipal Hospitals’ corporate and teaching facilities in India (IBM, 2015). Watson has the ability to read and understand natural language. As of December 2015, Watson for Oncology had ingested nearly 15 million pages of medical content, including more than 200 medical textbooks and 300 medical journals (IBM, 2015). Each month Watson ingests about 10,000 new scientific articles and data on 100 new clinical trials to keep up-do-date on new findings (Cavallo, 2017). A note of caution may be appropriate here. Watson for Oncology treatment recommendations are not based on its own insights from this data. Instead, they are based on training by physicians of Memorial Sloan Kettering Hospital who feed Watson information about how patients with specific characteristics should be treated (Ross & Swetlitz, 2017). Hence, its accuracy and overall value may be limited by differing medical practices and economic circumstances (Ross & Swetlitz, 2017). It may be fair to say that Watson for Oncology, while developing rapidly, is still in its early stage of development (Mearian, 2018).

As of 2015 there were 1 million new cancer cases diagnosed every year in India and this is expected to rise (IBM, 2015). In addition, India faces a shortage of oncologists, surgical oncologists and radiation therapists. Given the increasing number of cancer patients in India, fewer oncologists to treat them, the broad geographic footprint, and the rapid increase in scientific and clinical knowledge about care, physicians in India face a challenging time in staying up-to-date about best practices in treatment and care management. In this scenario, it was hoped that IBM Watson could help physicians in Manipal hospitals deliver the most advanced, effective, and cost-effective treatment to their cancer patients. According to a study published by Manipal Hospitals in 2018, Watson for Oncology was concordant with the hospital’s multidisciplinary tumor board in 93 percent of breast cancer treatment decisions (Somashekhar et al., 2018). However, showing that Watson agrees with the doctors only shows that it is competent in applying existing methods of care, not that it can improve them.

### AI’s impact on data privacy and ethical concerns

While looking to the future it would be remiss on our part not to assess the impact of AI on the privacy of medical and patient data. As the use of AI in medicine is increasing there is a corresponding increase in new threats to the privacy of healthcare related data (Redmore, 2019). A new study from the University of California, Berkeley suggests that progress in AI have rendered the privacy standards set by the Health Insurance Portability and Accountability Act of 1996 (HIPAA) obsolete (Na et al., 2018). It points out that removing healthcare data of identifying information doesn’t guarantee HIPAA compliance and this is a matter of concern. Specifically, it is now become possible due to AI to identify individuals by learning daily patterns from data collected by activity trackers such as smartwatches and smartphones, and then correlating it to demographic data (Redmore, 2019; Na et al., 2018). Per the authors, “machine learning successfully reidentified the physical activity data of most children and adults when using 20-minute data with several pieces of demographic information” (Na et al., 2018). The important conclusion is that privacy standards associated with the 1996’s HIPAA legislation need to be revisited and reworked such that the advances of AI and its impact on data privacy as it pertains to healthcare are factored in (Redmore, 2019; Na et al., 2018).

According to Anil Aswani, lead engineer on UC Berkeley’s privacy study, “You could imagine Facebook gathering step data from the app on your smartphone, then buying health care data from another company and matching the two. Now they would have health care data that’s matched to names, and they could either start selling advertising based on that or they could sell the data to others” (Redmore, 2019). This opens up a host of ethical concerns as well. HIPAA does not apply to tech companies that are at the forefront of advances in AI. It only protects patient healthcare data when it originates from organizations such as insurance companies and hospitals (Redmore, 2019).

It is reasonable to think that as AI makes it easier for companies to gain access to health data the likelihood of companies using it in unethical ways also increases. It may become possible for employers, credit card companies and others to use this AI generated data to discriminate based on pregnancy or disability status, for instance (Redmore, 2019; Na et al., 2018). Another potential example is cited in (Redmore, 2019) where genetics companies are selling customer data to pharmaceutical and biotech firms. The loophole here is that the service provided by companies of providing ancestry related information from DNA does not legally count as a healthcare service and so HIPAA privacy rules do not apply (Redmore, 2019). This opens the door for insurance companies to use use genetics data to make biased decisions as they pertain to insuring people and prices of insurance policy premiums (Redmore, 2019).

While AI has very promising applications in the AI field it is up to the users of AI to address some of these challenges to data privacy and related ethical concerns. We need to ensure that privacy policies and relevant ethical standards keep pace with the progress made by AI in medicine. Redmore (2019) makes some recommendations in this regard. Tech companies need to inform users on exactly how their data will be used and if the users so desire, they must have the option to decline consent to such use of their data (Redmore, 2019). Furthermore, tech companies must incorporate technical safeguards into their AI healthcare solutions to reduce the possibility of misuse and there must be a way to hold these companies accountable should they fail to do so (Redmore, 2019). Government must upgade legal frameworks such as HIPAA and/or create new ones as needed (Redmore, 2019).

## Assessing the Impact of AI on Physicians

In terms of predictive analytics and image recognition, AI may soon become more effective than physicians, who cannot handle millions of images in any reasonable timeframe. This has led to some concern that AI-based systems will replace physicians, especially radiologists. One narrative suggests that AI will interpret even the most complex clinical images as accurately as today’s most experienced radiologists and eventually replace radiologists (Pearson, 2017). The contrarian view is that this will not happen; rather AI will augment radiologists but not replace them (Pearson, 2017). There is yet another middle of the road view that at some point in the future AI will indeed replace radiologists, but it is not worth worrying about as that will happen in the distant future (Pearson, 2017). While it is difficult to project the impact of AI on radiologists in the future, Recht et al. provide a nuanced opinion in (Recht & Bryan, 2017a) that “AI will become a routine part of radiologists’ daily lives, making their work more efficient, accurate, and valuable”. AI-based machines will perform routine reading tasks such as quantification and segmentation and help free up radiologists to “perform more value-added tasks, such as integrating patients’ clinical and imaging information, having more professional interactions, becoming more visible to patients and playing a vital role in integrated clinical teams to improve patient care.” (Recht & Bryan, 2017a).

Looking beyond radiologists and considering physicians generally, it seems a reasonable prediction that AI are likely to augment physicians rather than replace them. There are several limitations of AI that lead one to this conclusion. AI cannot yet replace doctors at the bedside, given its limitations. Krittanawong (2018) points out that AI “cannot engage in high-level conversation or interaction with patients to gain their trust, reassure them, or express empathy, all important parts of the doctor–patient relationship.” Though one might speculate that this may change at some point in the future with AI being able to make a medical conversation. For example, Google demonstrated a phone call of its AI assistant in their Google I/O 2018 conference.

Physicians are still needed for traditional physical exams, especially in areas such as neurology, which require high-level patient-physician interaction and critical thinking (Krittanawong, 2018). Finally, even though AI may reach the point where it can conduct real-time CT scans or other physical scans, physicians will still be needed for interpretation in ambiguous and challenging cases. AI-based systems are based on precedence in the case of ML and DL, but such algorithms can underperform in novel or unusual cases of drug side effects or treatment resistance where there is no prior example to build on. For these reasons it can be concluded that AI-based systems will support the skills of physicians and are unlikely to replace the traditional physician–patient relationship.

## Conclusions

The avalanche of medical data in the form of clinical, genomic, and imaging data is only likely to accelerate as precision and personalize medicine matures. Consequently, for the foreseeable future medicine in the future medicine is likely to be even more data-dependent with the synergy between medicine and AI technology becoming more pronounced. In recognition of this important trend in modern medicine, medical schools are strengthening their emerging technology curricula. New courses are being offered by medical schools in technology infrastructure, ML, DL, and data management alongside their biology classes (Dyche, 2018).

AI will support the future needs of medicine by analyzing the vast amounts and various forms of data that patients and healthcare institutions record in every moment. AI is likely to support and augment physicians by taking away the routine parts of a physician’s work hopefully enabling the physician to spend more precious time with their patients, improving the human touch. While AI is unlikely to replace physicians in the foreseeable future, it is incumbent on medical professionals to learn both the fundamentals of AI technology as well as how AI-based solutions can help them at work in providing better outcomes to their patients. Or, it might come to pass that physicians who use AI might replace physicians who are unable to do so.

## Additional Information and Declarations

### Data Availability

The following information was supplied regarding data availability: No raw data or code was generated from this study; this article is a literature review.
